# The association of HIV-related stigma and psychosocial factors and HIV treatment outcomes among people living with HIV in the Volta region of Ghana: A mixed-methods study

**DOI:** 10.1371/journal.pgph.0002994

**Published:** 2024-02-29

**Authors:** Jerry John Nutor, Akua O. Gyamerah, Henry Ofori Duah, David Ayangba Asakitogum, Rachel G. A. Thompson, Robert Kaba Alhassan, Alison Hamilton

**Affiliations:** 1 Department of Family Health Care Nursing, School of Nursing, University of California, San Francisco, San Francisco, California, United States of America; 2 Department of Community Health and Health Behavior, University of Buffalo, Buffalo, New York, United States of America; 3 College of Nursing, University of Cincinnati, Cincinnati, Ohio, United States of America; 4 Language Center, College of Humanities, University of Ghana, Accra, Ghana; 5 Africa Interdisciplinary Research Institute, Accra, Ghana; 6 Centre for Health Policy and Implementation Research, Institute of Health Research, University of Health and Allied Sciences, Ho, Ghana; 7 VA Health Services Research & Development Center for the Study of Healthcare Innovation, Implementation & Policy, VA Greater Los Angeles Healthcare System, Los Angeles, California, United States of America; 8 Department of Psychiatry and Biobehavioral Sciences, David Geffen School of Medicine, University of California, Los Angeles, Los Angeles, California, United States of America; Weill Cornell Medicine, UNITED STATES

## Abstract

Stigma and discrimination have been identified as significant barriers to HIV treatment among people living with HIV (PLWH). HIV stigma affects decision to seek HIV testing and early treatment. Evidence shows that HIV stigma undermines antiretroviral therapy (ART) adherence by affecting the psychological process such as adjusting and coping with social support. In Ghana, stigma toward PLWH occurs in many ways including rejection by their communities and family members, ostracism, and refusal to engage in social interactions such as eating, sharing a bed, or shaking hands. Therefore. we examined PLWH’s experiences with different forms of HIV-related stigma and the impact on HIV treatment outcome in the Volta region of Ghana. We employed a convergent mixed-method approach consisting of a survey with 181 PLWH, four focus group discussions with 24 survey respondents, and in-depth interviews with six providers. We performed independent samples t-test, ANOVA, and chi-square test to test associations in bivariate analysis and analyzed qualitative data using thematic analysis. In all, 49% of survey respondents reported experiencing high internalized stigma, which was associated with high social support and depression (p<0.001). In qualitative interviews, anticipated stigma was the most salient concern of PLWH, followed by internalized and enacted stigma, which all negatively impacted HIV treatment and care. Stigma was experienced on multiple levels and affected psychosocial and treatment outcomes. Findings suggest urgent need for HIV-stigma reduction intervention among PLWH and their family, providers, and community members.

## Introduction

Stigma and discrimination have been identified as significant barriers to HIV prevention strategies [1–3]. HIV stigma is defined as irrational or negative attitudes, behaviors, and judgments toward people living with, or at risk of becoming infected with HIV [4]. Stigma may be enacted (experience of exclusion or discrimination), internalized (acceptance of negative attributes), or anticipated (expectation of future experiences of prejudice and stigmatizing behaviors) [5]. Internalized HIV stigma refers to validating negative feelings and beliefs associated with HIV and applying them to the self [5]. People living with HIV (PLWH) often think of themselves of acquiring a degraded characteristic and potentially endorse negative feelings and beliefs about their HIV status by comparing themselves to HIV-negative individuals [6, 7]. The process of confronting their past feelings and beliefs which may lead to internalized stigma. Consequently, internalized stigma may be a common reaction to acquiring HIV. However, anticipated stigma and enacted HIV stigma involve experiences with others.

HIV stigma presents serious challenges to HIV prevention and treatment globally, especially in sub-Saharan Africa. HIV stigma affects decision to seek HIV testing and early treatment [8, 9]. Evidence shows that HIV stigma undermines antiretroviral therapy (ART) adherence by affecting the psychological process such as adjusting and coping with social support [10]. One meta-analysis based on 64 studies found significant associations between HIV stigma and higher rates of depression, lower social support, and lower levels of adherence to ART and access to and usage of health and social services [11].

In Ghana, stigma toward PLWH occurs in many ways including rejection by their communities and family members, ostracism, and refusal to engage in social interactions such as eating, sharing a bed, or shaking hands [12]. For instance, in a national study conducted in 2014, stigma toward PLWH was assessed with questions about caring for PLWH, buying fruits from HIV-positive sellers, or allowing HIV-positive teachers to teach children. They found that only 8% of women and 14% of men reported positive to the questions on the HIV stigma indicators [13], indicating that HIV stigma is a major concern in fighting the HIV epidemic in Ghana.

In the past few years, Ghana has seen a decline in the HIV infection rate, which is attributed to the introduction and implementation of policies, programs, and strategic goals by the government of Ghana and other international bodies to end the HIV epidemic by 2030. One of these strategies is the introduction of the “Treat all” policy, which recommends ART for all PLWH regardless of their viral load or CD4 count [14]. The introduction of ART and the policy have transformed HIV infection from a nearly fatal disease to a manageable chronic disease. However, a major concern in Ghana is nonadherence to ART [15–17], with a recent study reporting adherence among PLWH at 42.9% [15].

Poor adherence to ART increases the risk of ART resistance and reduces treatment effectiveness toward viral load suppression, leading to disease progression, higher risk of death, and increased risk of viral transmission [18, 19]. HIV-related stigma may impact PLWH’s healthcare seeking behavior and poor ART adherence. Thus, this study aimed to examine PLWH’s experience with different forms of HIV-related stigma and their psychosocial and HIV treatment and care outcomes.

## Methods

### Study design and setting

The study employed a convergent mixed methods design. The study occurred in the Volta Region of Ghana, which has an HIV prevalence of 1.30% [20]. The region was chosen because of the high HIV prevalence rate. The study site was the Ho Teaching Hospital, which is the main referral ART clinic in the Volta Region of Ghana, with a total patient population of over 1,000 PLWH. For the quantitative phase, we conducted a descriptive cross-sectional survey with 181 PLWH clients at the Ho Teaching Hospital. For the qualitative phase, we conducted four focus group discussions (FGDs) with 24 survey respondents and in-depth interviews with six healthcare providers. See S1 Text for inclusivity in global research questionnaire.

### Recruitment and data collection

Using purposive sampling techniques, PLWH who receive care at the selected clinic were contacted in person by a nurse when they arrived for clinic visit to obtain their antiretroviral medication or for their regular appointment with their healthcare providers. The attending nurse discussed the study with potential participants. Those PLWH who agreed to learn more about the study were contacted by a trained research assistant. The research assistant explained the purpose, benefits and risks, and confidentiality to potential participants. A copy of the study’s information sheet was given to the potential participants who could read. For the PLWH who could not read, study personnel explained the information sheet in a local language that they could understand. Those who agreed to participate in the study were given a consent form to sign or thumb print if they could not sign. PLWH were eligible to participate if they were age 18 years or older, enrolled in ART for at least 6 months, and identified by clinic staff as either a high or low clinic attendee. We enrolled participants who were on ART for at least 6 months because previous studies have shown that adherence to ART decline among PLWH after the first six months [21, 22]. High or low clinic attendees were recruited to ensure diverse sample. We excluded PLWH who were seriously ill, defined as hospitalization for any reason in the past one month. Participants received $10 compensation after the interview for their time and travel cost. The survey questionnaire was self-administered by participants who could read and those who could not read were surveyed through a survey administrator. Participants who participated in the survey data collection were also asked if they would be interested in a focus group discussion later. Those who agreed were contacted after the quantitative data collection. Data was collected between April 30 and August 31, 2021.

### Survey measures

**Stigma** was evaluated with the Internalized Stigma of HIV/AIDS Tool (ISAT) [23]. The ISAT tool is a 10-item scale that assesses the negative self-perceptions of individuals regarding HIV. The items on the ISAT are evaluated on a five-point Likert scale ranging from 1 (strongly disagree) to 5 (strongly agree). High total ISAT score is indicative of a high level of internalized stigma. In the bivariate analysis, the ISAT scores were categorized based on the median score of the sample (median = 27). Therefore, low stigma was defined as ISAT scores below the median score of 27 whereas high stigma was defined as ISAT scores greater than or equal to 27. Inter-item reliability test of the ISAT tool in our sample revealed a Cronbach’s alpha value of 0.85.

**Depression** was evaluated with the Center for Epidemiologic Studies Depression Scale (CES-D) [24]. The CES-D consist of 20-item Likert type questionnaire that assesses the number of days in the past week during which persons felt depressed. The response options for each item range from rarely or none of the time (score = 0) to most or all the time (score = 3). The CES-D scores range between 0 and 60 and high scores are suggestive of a greater risk for symptoms of depression. Inter-item reliability analysis for our sample data revealed an acceptable Cronbach’s alpha (0.87). Depression scores were categorized using the cutoff point of 16: individuals with scores less than 16 were defined as having a low risk of depressive symptoms, whereas scores ≥ 16 were categorized as high risk of depressive symptoms [25].

**Social Support** was measured with the Interpersonal Support Evaluation List-12 (ISEL-12) [26]. The ISEL-12 tool comprises 12 items rated on a 4-point Likert scale that assesses perceived social support by asking respondents about their ability to find assistance in various social conditions of need. The scores of the responses range from 1 (definitely false) to 4 (definitely true*)*. Six items were reverse scored (Items 1, 2, 7, 8, 11, and 12). High ISEL-12 total scores indicate a high level of perceived social support. The ISEL-12 has three subscales including Appraisal, Belonging, and Tangible Support domains, with a higher score under each domain indicating higher levels of perceived social support. We performed an inter-item reliability test for the overall ISEL-12 scale for our sample, and we found an acceptable Cronbach’s alpha value of 0.82.

**Adherence** was measured adherence using the simplified medication adherence questionnaire (SMAQ) [27]. The SMAQ tool consist of 6 items: Item 1: *Do you always take your medication at the appropriate time*? *Item 2*: *When you feel bad*, *have you ever discontinued taking your medication*? *Item 3*: *Have you ever forgotten to take your medication*? *Item 4; Have you ever forgotten to take your medication during the weekend*? *(Item 1–4 are scored as*: *0 = No or 1 = Yes); Item 5*: *In the last week*, *how many times did you fail to take your antiretroviral drug*? (1 = Never,2 = 1–2 times,3 = 3–5 times, 4 = 6–10 times,5 = More than 10 times; Item 6: *Since your last visit*, *how many whole days have gone by in which you did not take your medication*? (Open ended). Item six was subsequently categorized as ≤2days or >2days. A person was categorized as non-adherence if any of these criteria were met: a negative response to item 1, *OR* a positive response to either item 2, 3, or 4, *OR* >2 doses missed in the past week, *OR* >2days of non-medication in the past week. We performed an inter-item reliability test for the SMAQ tool for our sample, and we found a Cronbach’s alpha value of 0.61.

**Demographic and other variables** included relationship status (levels: single/widow; in current relationship), residence (rural; urban), gender (male; female), age, level of education (levels: No formal education; Primary; Secondary or Post-Secondary), monthly income (levels: <1000 Cedis; ≥1000 Cedis), and disclosure of HIV status to partner (yes; no), and disclosure to anyone (yes; no) (Table 1). Alcohol use was measured as drinking more than one glass per week.

**Table 1:** Sample characteristics from survey (N = 181).

Variable	Frequency (%)^a^
**Gender**	
Female	25 (13.8%)
Male	101 (55.8%)
Missing	55 (30.4%)
**Age**	
Under 30	15 (8.3%)
30–39	35 (19.3%)
40–49	55 (30.4%)
50–59	45 (24.9%)
60+	25 (13.8%)
Missing	6 (3.3%)
Mean (SD)	46.5 (12.4)
**Marital Status**	
Single/Widow	79 (43.6%)
Relationship	95 (52.5%)
Missing	7 (3.9%)
**Level of Education**	
No Formal education	18 (9.9%)
Primary	86(47.5%)
Secondary	49(27.1%)
Post-Secondary (undergraduate, masters and doctorate)	21(11.6%)
*Missing*	7(3.9%)
**Monthly Income**	
<1000 Cedis	135 (74.6%)
≥1000 Cedis	25 (13.8%)
Missing	21 (11.6%)
**Place of Residence**	
rural	86 (47.5%)
urban	90 (49.7%)
Missing	5 (2.8%)
**Disclosure to Partner**	
Yes	84 (46.4%)
No	85 (47.0%)
Missing	12 (6.6%)
**Disclosure to anyone**	
Yes	112 (61.9%)
No	61 (33.7%)
Missing	8 (4.4%)
**Alcohol**	
No	108 (59.7%)
Yes	65 (35.9%)
Missing	8 (4.4%)
**Use hard Drugs**	
No	169 (93.4%)
Yes	3 (1.7%)
Missing	9 (5.0%)
**Depression (CES-D 20) Total**	
Mean (SD)	9.1 (8.8)
** Depression status (CES-D 20)**	
0–15 score: No depressive symptom	133 (73.5%)
16 and above: Depressive symptom	37 (20.4%)
Missing	11 (6.1%)
**ISEL12 TOTAL**	
Mean (SD)	18.7 (6.6)
**Social support status (ISEL12)**	
less than median score of 17: weak I-support	83 (45.9%)
From the median score of 17: strong I-support	87 (48.1%)
Missing	11 (6.1%)
**ISAT Total**	
Mean (SD)	26.4 (8.6)
**Internalized stigma status (ISAT)**	
less than median score of 27: low stigma	82 (45.3%)
From the median score of 27: high stigma	88 (48.6%)
Missing	11 (6.1%)
**Adherence Status (SMAQ)**	
Adherent	54 (29.8%)
Non-Adherent	105 (58.0%)
Missing	22 (12.2%)

^a^ Categorical variables are reported as frequency (%)

continuous variables are reported as mean (SD)

### Focus group discussions and in-depth interviews

We conducted four FGDs with a sub-sample of 24 survey respondents. FGDs consisted of 5 to 7 people each. FGD participants were purposively sampled to generate a sample that was diverse in age, gender, residency, and years since HIV diagnosis. FGDs occurred in a private room at the study site and were facilitated by two trained members of the research team. FGDs were conducted in English and Ewe and lasted 60 to 90 minutes. In all, we conducted four FGDs two with all females, one with all males, and one with mixed gender). PLWH participants were asked questions about their experiences with getting HIV treatment and accessing care and with taking antiretroviral medication; factors that make it easy or difficult to access treatment and care and take their medication; and whether they have disclosed their HIV status to someone in their life. They were also asked questions about different types of HIV treatment and care support interventions (peer and social support, mental health support, provider support, and treatment access interventions) and whether and why they would be interested or not in these types of interventions.

We also conducted in-depth interviews with six HIV service providers (three medical doctors and three nurses) who provide HIV care and counseling services because the health facility lacks clinical psychologists and/ or straight counselors. The interviews were conducted in English over Zoom by AOG which lasted about 60–80 minutes. Providers were asked questions about their PLWH clients’ experiences with getting HIV treatment and accessing care and with taking antiretroviral medication, as well as factors that make it easy or difficult for clients to access treatment and care and take their medication.

### Quantitative data analysis

Univariate and bivariate analyses were performed. Frequencies and percentages were reported for categorical variables while mean and standard deviation were reported for continuous variables in the univariate and bivariate analyses. Independent samples t-test, one-way analysis of variance (ANOVA), and chi-square test for independence were used to test association in bivariate analysis. The analytical sample for the univariate and bivariate analysis were 181, and 170 respectively. In multiple logistic regression estimates of factors associated with stigma, depression was excluded due to multicollinearity. Missing variables were excluded in the multivariable analysis. Statistical significance was pegged at alpha level of 0.05. All analysis was performed in Stata 14 software [28].

### Qualitative data analysis

All interviews were audio recorded and transcribed by members of the research team. The transcript that were in Ewe, a widely spoken language in the Volta region were translated to English by a member of the study team. All interview transcripts were closely reviewed for accuracy and inconsistencies were corrected.

We analyzed transcripts using thematic analysis [29]. We developed an initial codebook of a priori codes based on the focus group and in-depth interview guide topics. Two members of the research team (DAA and AOG) conducted qualitative data analysis. The coders each independently coded a FGD and an in-depth interview transcript. Coders then discussed and compared the coding and resolved differences. The codebook was revised after this step and the primary coder (DAA) completed coding the remaining transcripts. We further analyzed “HIV stigma,” “enacted stigma,” “internalized stigma,” and “anticipated stigma” data segments for emergent patterns and themes. We summarized the analysis for each group and displayed themes on a table to compare differences and similarities between the groups. We conducted data analysis using Dedoose desktop version 9.0.17 [30].

While our quantitative data provides findings on internalized HIV stigma, qualitative analysis of stigma data segments offers in-depth and complementary findings on various forms of stigma clients experienced or anticipated due to their HIV status.

### Ethics statement

The study was conducted in accordance with the Declaration of Helsinki for research involving human subjects. Ethical approval for the study was obtained from the University of Health and Allied Sciences (UHAS) Research Ethical Committee with reference number UHAS-RECA.6 [1] 20–21 and the University of California, San Francisco Institutional Review Board with reference number 20–32955. Permission was also sought from management of the Ho Teaching Hospital and the HIV Clinic. Confidentiality was ensured at all stages of the process. Informed consent was obtained from the participants before the interview. Respondents gave written informed consent prior to enrollment using the approved consent forms. Respondents were assured that refusal to participate or withdrawal from the study would not affect their access to healthcare services at the clinic.

## Results

### Sociodemographic characteristics

A total 181 participants were included in the study. A majority (56%) were male and about half of the participants were urban residents. About 53% of the participants were in a relationship at the time of the survey and 44% were single/widowed. The mean age was 46.5±12.4 years. Approximately 48% of participants had primary school education, 27.1% had high school education, and 11.6% had attained post-secondary education. A majority (75%) earned less than GH1000 (approximately 100 USD) monthly income. In terms of disclosure of HIV status, 47% had not disclosed their status to their partners and 34% had not disclosed to anyone. The prevalence of alcohol use was 35.9% and only 1.7% prevalence of other hard drug use such as marijuana and cocaine.

The average internalized stigma score was 26.4 ± 8.6 and approximately 49% reported experiencing high internalized stigma. The mean depression score was 9.1±8.8 with 20.4% reporting depression symptoms. The average total social support score was 18.7±6.6 with about 46% reporting low social support. The ART non-adherence rate was 58.0% on the SMAQ tool (Table 1).

### Bivariate associations of internalized stigma

Table 2 displays findings on bivariate associations between internalized stigma and sociodemographic, psychosocial, and ART variables. Among participants who reported low internalized stigma, 26.8% reported high social support while among those with high internalized stigma, 73.9% reported high social support (p<0.001). None of those who had low internalized stigma reported depression symptoms while 42.0% of those who reported high internalized stigma reported depression symptoms (p<0.001). The non-adherence rate was 67.1% among participants with low stigmatization and 56.8% among participants with high stigmatization (p = 0.04). No significant association was found between stigma and the following variables: gender, age, marital status, place of residence, income, disclosure status, use of alcohol /drugs, and attitudes towards ART.

**Table 2:** Sample characteristics by stigmatization (N = 170).

	Low internalized stigma (score less than 27)	High internalized stigma (Score 27 and above)	Total	p-value
	(N = 82)	(N = 88)	(N = 170)	
**Gender**				0.960
Female	11 (13.4%)	14 (15.9%)	25 (13.8%)	
Male	45 (54.9%)	56 (63.6%)	101 (55.8%)	
*Missing*	26 (31.7%)	18 (20.5%)	55 (30.4%)	
**Age**				
Mean (SD)	47.5 (13.4)	45.8 (11.8)	46.6 (12.6)	0.376
**Age Categories**				0.979
Under 30	7 (8.5%)	8 (9.1%)	15 (8.3%)	
30–39	15 (18.3%)	19 (21.6%)	35 (19.3%)	
40–49	25 (30.5%)	27 (30.7%)	55 (30.4%)	
50–59	22 (26.8%)	22 (25.0%)	45 (24.9%)	
60+	13 (15.9%)	12 (13.6%)	25 (13.8%)	
*Missing*	0 (0.0%)	0 (0.0%)	6 (3.3%)	
**Marital Status**				0.969
Single/Widow	37 (45.1%)	39 (44.3%)	79 (43.6%)	
Relationship	45 (54.9%)	48 (54.5%)	95 (52.5%)	
*Missing*	0 (0.0%)	1 (1.1%)	7 (3.9%)	
**Place of Residence**				0.446
rural	43 (52.4%)	41 (46.6%)	86 (47.5%)	
urban	39 (47.6%)	47 (53.4%)	90 (49.7%)	
*Missing*	0 (0.0%)	0 (0.0%)	5 (2.8%)	
**Monthly Income**				0.657
<1000 GH Cedis	68 (82.9%)	64 (72.7%)	135 (74.6%)	
≥1000 GH Cedis	13 (15.9%)	10 (11.4%)	25 (13.8%)	
*Missing*	1 (1.2%)	14 (15.9%)	21 (11.6%)	
**Disclosure to Partner**				0.278
Yes	44 (53.7%)	38 (43.2%)	84 (46.4%)	
No	38 (46.3%)	46 (52.3%)	85 (47.0%)	
*Missing*	0 (0.0%)	4 (4.5%)	12 (6.6%)	
**Disclosure to anyone**				0.638
Yes	55 (67.1%)	56 (63.6%)	112 (61.9%)	
No	27 (32.9%)	32 (36.4%)	61 (33.7%)	
*Missing*	0 (0.0%)	0 (0.0%)	8 (4.4%)	
**Alcohol**				0.773
No	53 (64.6%)	55 (62.5%)	108 (59.7%)	
Yes	29 (35.4%)	33 (37.5%)	65 (35.9%)	
*Missing*	0 (0.0%)	0 (0.0%)	8 (4.4%)	
**Use hard Drugs**				0.595
No	81 (98.8%)	85 (96.6%)	169 (93.4%)	
Yes	1 (1.2%)	2 (2.3%)	3 (1.7%)	
*Missing*	0 (0.0%)	1 (1.1%)	9 (5.0%)	
**Depression (CES-D 20) Total**				
Mean (SD)	3.5 (3.7)	14.4 (8.9)	9.1 (8.8)	<0.001
**Depression status (CES-D 20)**				<0.001
0–15 score	82 (100.0%)	51 (58.0%)	133 (73.5%)	
16 and above	0 (0.0%)	37 (42.0%)	37 (20.4%)	
*Missing*	0 (0.0%)	0 (0.0%)	11 (6.1%)	
**ISEL12 Total**				
Mean (SD)	15.5 (5.2)	21.6 (6.4)	18.7 (6.6)	<0.001
**Social support status (ISEL12)**				<0.001
less than median score of 17: low support	60 (73.2%)	23 (26.1%)	83 (45.9%)	
From the median score of 17: high support	22 (26.8%)	65 (73.9%)	87 (48.1%)	
*Missing*	0 (0.0%)	0 (0.0%)	11 (6.1%)	
**Adherence Status (SMAQ)**				0.040
Adherent	19 (23.2%)	35 (39.8%)	54 (29.8%)	
Non-Adherent	55 (67.1%)	50 (56.8%)	105 (58.0%)	
*Missing*	8 (9.8%)	3 (3.4%)	22 (12.2%)	

Categorical variables are reported as frequency (%)

continuous variables are reported as mean (SD)

### Multivariable associations of internalized stigma

The multivariable analysis revealed that high social support was associated with 15 times greater odds of having high internalized stigma, after adjusting for gender, age, marital status, place of residence, monthly income, and disclosure to partner (OR:15.06,95% CI: 5.32–42.63, p<0.001). All other predictors were not statistically significant (Table 3).

**Table 3:** Multivariable estimates of associations for HIV stigma (N = 170).

Predictors	OR	[95% Conf Interval]		p-value
		Lower	Upper	
**Social support status (ISEL12)**				
low support	Ref.	-	-	-
high support	15.06	5.32	42.629	<0.001
**Gender**				
Female	Ref.	-	-	-
Male	0.416	0.121	1.437	0.166
**Age**	0.992	0.955	1.031	0.694
**Marital Status**				
Single/Widow	Ref	-	-	-
Relationship	1.589	0.519	4.864	0.417
**Place of Residence**				
rural	Ref.	-	-	-
urban	1.603	0.639	4.02	0.315
**Monthly Income**				
<1000 GH Cedis	Ref.	-	-	-
≥1000 GH Cedis	0.883	0.231	3.37	0.856
**Disclosure to Partner**				
No	Ref.	-	-	-
Yes	1.559	0.524	4.637	0.425

### Qualitative findings on HIV-related stigma

In focus group discussions with PLWH and in in-depth interviews with service providers, anticipated stigma emerged as the most salient concern of PLWH clients, followed by internalized and enacted stigma. Table 4 provides participant characteristics from the qualitative phase. Their experiences, anticipation, and internalization of stigma occurred on multiple levels. The sections below describe the forms of stigma they experienced, the context (site) and source (actors) of those stigmas, on what levels they experienced this stigma (i.e., individual, interpersonal, community, organizational, societal), and reasons or catalyst for the stigma. We also discuss the impact of these forms of stigma on clients’ lives and their HIV care-seeking behavior. See Table 5 for types, sites, sources, level, context, and impact of stigmas reported and Table 6 for participant excerpts on forms of stigma reported.

**Table 4:** Participant characteristics from qualitative phase.

PLWH focus group discussions (N = 24)				
PLWH Client	Gender	Age (Years)	Occupation	Years since diagnosis
1	Female	42	Trader	9
2	Male	72	Artisan	7
3	Female	46	Head dresser	13
4	Female	52	Trader	9
5	Male	64	Security man	10
6	Female	38	Trader	7
7	Female	38	Pure water hacker	12
8	Male	49	Farmer	14
9	Female	43	Trader	12
10	Female	42	Trader	11
11	Female	47	Trader	18
12	Male	64	Farmer	11
13	Male	72	Pensioner	6
14	Female	31	Seamstress	3
15	Male	56	Security man	11
16	Male	66	Security man	7
17	Male	60	Trader	2
18	Male	47	Driver	5
19	Male	57	Farmer	7
20	Female	39	Seamstress	6
21	Female	62	Unemployed	15
22	Female	44	Trader	14
23	Female	58	Farmer	14
24	Female	52	Trader	8
**Provider in-depth interviews (N = 6)**				
**Provider**	**Gender**	**Age (years)**	**Occupation**	**Years in position**
HCP 1	Female	30	Nurse	7
HCP 2	Male	26	Nurse	1.5
HCP 3	Male	39	Nurse	2
HCP 4	Female	38	Medical doctor	3
HCP 5	Female	29	Medical doctor	1
HCP 6	Male	54	Medical doctor	7

**Table 5:** Types, source, level, and context of stigma experienced and their impact on PLWH.

Types of Stigma	Site and source of stigma (where/who are they experience or anticipate stigma)	Level of stigma (individual, community, organizational, etc.)	Reasons or catalyst for stigma (why they experience, internalize, or anticipate stigma)	Impact of stigma
**Anticipated**	**Where**: Clinic, community settings, HIV programs (i.e., PLWH group or community-based interventions) **Source:** Acquaintances, friends, family, and peers; community members; healthcare providers/staff	Interpersonal Community Organizational	Fear of HIV status being disclosed due to being seen at ART clinic by someone they know or due to participating in HIV programs in their community or with other people. Fear HIV status will be used against them or cause gossip if they disclose or their status is disclosed.	1. HIV status non-disclosure 2. Avoid clinic/delay decision to access HIV treatment 3. Travel far for HIV care and treatment services in other districts 4. Disinterest in ART home or community delivery services/interventions
**Enacted**	**Where**: Family, community, clinic **Source:** Family members, community members, healthcare workers/staff	Interpersonal Community Organizational	Experiencing HIV stigma due to physical markers of being sick such as losing weight. Disclosure of status by health workers. Stigmatizing gossip by those who know of HIV status. Abandonment of family due to HIV status.	1. Emotional and mental distress 2. HIV status non-disclosure 3. Social isolation 4. Perception of death of PLWH 5. Avoid clinic/delay decision to access HIV treatment
**Internalized**	**Where:** Self/PLWH **Source**: Individual, community members, societal views, clinic staff, healthcare providers	Individual Community Organizational Societal	Internalizing HIV stigma due to societal views of PLWH as inferior. Denial of and inability to accept HIV diagnosis. Enacted stigma such as gossip, abandonment, isolation, shame.	1. Avoiding marriage due to HIV status 2. Avoiding people 3. Avoid clinic in fear of encountering them at the ART clinic 4. Discouragement 5. Delay linkage to ART

**Table 6:** Excerpts of forms of stigma, HIV disclosure, and social support reported by people living with HIV and impact.

THEMES	QUOTES
**ANTICIPATED STIGMA**	
	FGD2, R7 “In our community, the people are all gossip. They will see you entering the [clinic] room and will find out by all means what you actually went to do there. The question is what you are also searching for that you saw your sister there. What they don’t see is what they will say they have seen.”
	FGD 4, R1 “Maybe he also came here with the same disease, but some of us we talk so much. Maybe when he meets you here, he goes to tell his friend or someone in town and maybe something happens one day then the person used it to insult you. So when I’m coming, I think about it that no known person should meet me on the way, but when I get home I feel a bit at ease.”
	FGD 3, R3 “The drugs should be at the facility here. When we need it, we will follow up and come for it. This is because if they want to bring it to your house and they don’t meet you, they will just give it to someone and that’s it. Then the households will go and spread [that you have HIV]. So, it should be [at clinic] for us just as we have been coming for it.”
**ENACTED STIGMA**	
	FGD 4, R2 “So when people come to see me then they’ll say she has AIDS. Then they sent my name to town that I have AIDS. Some people came and said they heard I have AIDS and I said you are saying it.”
	FGD 2, R1 “Someone from the hospital called one of our sisters working at municipal hospital that this and this is happening to my brother and the wife. They spread the information around. So, it’s not [just] people outside who spread the information, but insiders also spread it. We are not the only ones spreading the information, but health workers are also sending the information out.”
	FGD 1, R4 “It’s like some of us talked about it previously that, excuse me to say, some of the health workers carry information from here [clinic] outside. If those [providers] who will be with us will go through proper training that they should never carry our information outside. But without them going through any training, then for me as an individual, I don’t like it.”
**INTERNALIZED STIGMA**	
	FGD 2, R4 “So, I decided that I won’t marry again because I don’t know where I will go that they will embarrass me for the second time. So, I careless about marrying again and consoled myself that after-all, I have two younger brothers that I should take care of and when they grow up, they will come and look after me. So when I came here in the 1st to 2nd years, they came to counsel me that it shouldn’t go that way”.
	FGD 2, R2: “You see, we are the cause of discrimination. You wake up going to take your medication and where you are going is known to only you. But you will turn looking at your back. Who are you looking at in your back? You wanted to see if any of your family member is there. You just have to forget anything because we all came to hospital, and you came for paracetamol and I also came for paracetamol. Who knows? So, we are the ones causing it. We are the cause of people talking about us because when you come to the hospital, you only know what you have come to do, and you will disclose to someone before the person will know.”
	FGD 2, R6: “It is good we do this program because it will let people living with the disease be encouraged. There are people living with the disease but because of shame and stigma, they pretend they don’t have it unless they are sick and come to the hospital.”
**IMPACT OF STIGMA**	
AVOID CLINIC/CARE	HCP 3: “Let’s say if today is the clinic day, they are supposed to come today as a clinic day, they know a lot of people will come around. That day they will not come.”
Delay ART initiation/HIV care	HCP 4: “What happens is that some people because of stigma, some [PLWH] do not come early. They become sick because they are diagnosed; when they come very sick through the emergency and are on admission, by that time, most of them have lost ability to work”.
HIV status non-disclosure	FGD 4, R4: “What I also thought is that a lot of people don’t want people to know that they are living with the disease. That is where the problem is. If you should know or the person tells you, that is the only way that you will know. But because of our own plenty talks, the person may not disclose it to you. So many of the things that makes it difficult for people living with this disease is from too much talk. If not that, it’s not anything.”
Long distance travel for HIV care	HCP 4: “Then there is one thing…because of stigma, some the patients do not take treatment at the locality where they are working or where they are living. They prefer to go to another Centre where they are not known in that community to take services. So, for us, people from Accra or Eastern region are willing to come and take the treatment at our place.”
PERCEPTIOn of death	FGD 1, R2 “So I advise people like that. I saw a sister that I met in this room and advised her on how she is carrying out herself that am not pleased with, one of her siblings who brought her and got to know [her HIV status], it has turned to quarrels among [their family] in the house…She then left to [another town] and she got there not long, she died. If she was to listen to me, am sure she would have been alive today.”
resilience against stigma	FGD 4, R3 “Me, before the break of day for my appointment it doesn’t worry me because I am bold that I can stand and do everything. I don’t have fears that I’ll come here and meet someone. We’re all traders. When you go to meet your friend in the market then that’s it, but if you don’t meet him too that’s it. So, coming here doesn’t worry me”. FGD 4, R2 “I have been saying that when you’re drumming following me that I have HIV, I won’t mind you. You who’s drumming is the one people will see. Because there are so many diseases in the world. So, if you see your friend and you’re pointing a finger at him do you know which sickness you’ll also contract tomorrow?”
**HIV DISCLOSURE & SOCIAL SUPPORT**	
Emotional & INSTRUMENTAL support	FGD 2, R2: “[The nurse] just called us to come and immediately we got to the room, she said, you have HIV. She just said it openly. So, I decided to run away, and my man asked where am going. He told me that people are living with it and am not the first person so I should calm down. After we left, he was consoling me at home the whole night. He was consoling me to the extent that he called the nurse and asked her if he can have sex with me…so that I will be comforted and the nurse said oh, he can do it, but he should protect himself because this is what they detected. He agreed and we have been living since.” FGD 4, R4, “Ooh [HIV illness] became serious, so when they sent me to the hospital, they themselves told the [family] who sent me that this is the issue with me. So [family] received me fine. They didn’t discriminate. So, at the time when I couldn’t do anything, but they did it until I recovered.”
No Stigma & discrimination	FGD 2, R1: “For support, I told my family including my children. When I was diagnosed, I first told my family. Myself and siblings are ten. When the thing happened several years ago, if I could remember well, they were the first people they disclosed it to, but they supported me. Because of that, if am at work or coming here for the medication, they remind me of it. I’ve not come across stigma. They received me well.” FGD 2, R5: “All my children supported me. 4 boys and 1 girl all supported me. Unfortunately, my wife is also affected, and we are both going through. So, there is no discrimination in my home.”
Treatment and Care support	FGD 2, R2: “When I came home, I sometimes pretend to forget, and [my husband] will ask me if I’ve taken the drug. Even when he’s on [work] operation outside the country, he will call me and ask if I’ve taken the drug…He is very supportive. He’s done very well.” FGD 2, R6: “Because of my condition, any kind of work that I do, [my family] tries to support me. If I forgot to take my medication, my children or husband do remind me.” FGD 4, R2 “But in my case, I come for my drug and get home before my mother gets to know. Then she’ll ask, ‘Have you gone for your medication already?’ I’ll respond yes and show it to her. She’ll then say I should take good of it. Have you taken it? It’s 8 o’clock already o and I’ll say I’ve taken it already. She’ll say as for today you have taken it early. So, all that makes me not to harbor anything in my mind.” FGD 4, R4: Taking the medication is the real deal. Some of my siblings are not in [local town] but they call from time to time. Are you still going for your medication? Are you taking it on time? And what you’re not supposed to do. When you’re on medication what you’re told not to do.

### Anticipated stigma

The most common form of stigma reported by FGD participants was anticipated stigma. Anticipated stigma was defined as fear a person has that they will experience stigma, prejudice, and/or discrimination due to their HIV status [5]. Clients’ anticipated stigma were shaped by their experiences of enacted stigma, as well as by internalized stigma. Clients anticipated stigma on the interpersonal, community, and organizational levels. They anticipated stigma at sites like the clinic, community settings, and at HIV programs/interventions. They also anticipated stigma from people they are acquainted with, including friends, family members, peers with HIV lived experiences, community members, and healthcare providers and staff.

Clients commonly anticipated stigma at the site of HIV care and in their community. In particular, clients expressed fear that their HIV status would be disclosed due to being seen by someone they know at an ART clinic. Several shared that they were concerned that those who learn of their status would gossip, especially on the community level, and draw negative attention and stigma towards them. One participant shared that some PLWH had their status disclosed by a clinic worker; thus, due to that incident and having family that work at the clinic, she was very careful about when she seeks care to avoid having her status known and disclosed:

“I have two of my sisters working in the hospital here. So, when I’m coming here, I’m scared that no one should see me. When I want to come here, I wake up very early so that no one will see me and when I want to leave, I leave very early.” (FGD2, R1)

Many healthcare providers corroborated clients’ concerns about experiencing stigma at the site of care. A nurse, for example, shared that some clients who feel or perceive stigma do not come to the clinic in fear of encountering someone they know: “Some of them, they feel stigma and they stay there that they don’t want to come and see who they don’t want, maybe they might know somebody and they will come and meet the person [at the clinic].” (HCP 3)

Additionally, in discussions about the types of HIV interventions that clients may find useful towards improving their HIV care outcomes, some clients shared that they were concerned about interventions that may disclose their HIV status to other people or community members, like PLWH group support interventions, or interventions where HIV treatment and care are delivered in a community site or at home. As one client shared in response to an intervention program that would deliver antiretrovirals at a community site of the client’s choosing: “We don’t want drugs to be distributed to us in our homes for people to identify us.” (FGD4, R2)

### Enacted stigma

Clients reported enacted stigma on the interpersonal, community, and organizational levels from family and community members, as well as from healthcare workers and staff in the clinical setting.

Clients reported experiencing HIV stigma due to physical markers of being sick such as losing weight due to their HIV infection. As one participant shared about her experience, some people she knew talked about her having HIV due to her physical appearance:

“When people come to see me then they’ll say she has AIDS. Then they sent my name to town that I have AIDS. Some people came and said they heard I have AIDS…and I said, ‘Ah, you are saying that nobody fell sick in this town and became this thin than this my AIDS before?” (FGD 4, R2)

A key issue of concern among clients and service providers was that some healthcare workers have disclosed clients’ HIV status, indicating violations of confidentiality and issues of professionalism at the site of care (see Table 6 for examples). In these cases, workers in the clinical setting, whether providers or staff, reportedly discussed or gossiped about the HIV status of clients inside and outside of the clinic, some of which clients became aware of through experienced stigma.

Another form of enacted stigma was related to the consequences of one’s status being disclosed, reflecting the concerns those who anticipated stigma expressed. In particular, some participants reported being gossiped about or knowing PLWH who were gossiped about by people who learned of their HIV status, as well as experiencing conflict with friends and abandonment from family members due to disclosure of their HIV status.

### Internalized stigma

The third form of HIV stigma clients reported was internalized stigma. Internalized stigma was experienced from oneself and other PLWH and the sources of the beliefs and experiences being internalized were on the individual, community, organizational, and societal from personal relationships, community members, societal views, and clinic workers.

Several participants shared or acknowledged that much of the anticipated stigma PLWH experience is due to internalized stigma. For these participants, internalized stigma makes PLWH worry about prejudice or discrimination. For example, one client (FGD 1, R4) discussed how a support program for PLWH clients was dissolved because “we faced a lot of problems with people as some people said that if they come for a meeting that is called, they won’t attend because some people will just want to come and spy on them.” They added that similarly, some PLWH avoided a nutrition support program for clients due to worry that program staff would know their HIV status.

“Because of our own discouragement that people will see us and assume that these are the kind of people having the disease, they won’t accept to come [to HIV food support program]. Meanwhile it is not like that. People don’t know that these are the kind of people coming to take food from him. But within themselves [PLWH], they were already discouraging themselves. When they call them, they will not come [to program].” (FGD 1, R4)

### Impact of stigma

Notably, the three forms of stigma participants reported had tangible impacts on the lives and health-seeking behavior of clients (Table 6). Some participants avoided people in the community or at the clinic due to experiencing, internalizing, or anticipating stigma. Others structured their HIV care-seeking routine/strategy to avoid encountering someone they know at the clinic, including traveling long distances to other cities/towns to get care. These forms of stigma also affected acceptance of HIV diagnosis and timely initiation of HIV treatment and care. One nurse shared that a client she buys products from in the market who encountered her at the clinic avoided returning to the clinic due to stigma:

“The day that she came and saw me at the HIV unit, she never came back there. It’s like she feels shy to come so because of that me too I feel shy to go to her and buy again because she saw me at the place that she was coming for the drug.” (HCP 3)

In addition, experiencing or anticipating stigma also affected PLWH’s interest or participation in HIV-related interventions that would help improve care outcomes, such as nutritional support, group support, treatment access support, or disclosure support programs.

In terms of psychosocial and personal impact, experienced, anticipated, and internalized stigma also caused PLWH to feel discouraged, emotionally and mentally distressed, socially isolated, and avoidant of marriage and intimacy. There was even a perception that enacted stigma and subsequent isolation from family lead to death among PLWH death due to avoiding receiving care (See Table 6 for excerpts). Some participants also avoided disclosure of status to family members and friends due to anticipated or experienced stigma. As one client shared, they did not want to disclose their HIV status in fear of it drawing gossip and causing further stigma.

“Because of stigmatization, that is why nobody wants to expose himself. The few who know that I’ve acquired the virus are limited to one to three people in the community. But if you should expose it right now, they will be in insulting you and you will feel inferior in the society.” (FGD 2, R4)

### Resilience against stigma

Although many clients discussed stigma affecting their lives and decisions, some clients acknowledged that while HIV stigma was an issue in the community and in their lives, they were resilient against this stigma. These clients did not let or want stigma to halt their HIV care- seeking, change their routines, and/or discourage them. As one client (FGD 4, R4) shared, “People may talk but I don’t care because it’s my life that I’m after, so I don’t mind them.”

These participants expressed concern that they felt that experienced stigma and their internalization/anticipating were negatively affecting the health and lives of PLWH, and unnecessarily. As one participant (FGD 2, R6) shared, “There are people living with the disease but because of shame and stigma, they pretend they don’t have it unless they are sick and come to the hospital.”

### Disclosure, social support, and treatment and care

While HIV stigma impacted whether clients disclosed their HIV status, many participants shared that they had disclosed their HIV status to someone, mostly family members, or in a few instances, a healthcare provider disclosed their status to a family member with their consent or out of medical necessity. Family members they disclosed to were usually their spouse, some of whom were also living with HIV, siblings, parents, and children. Some clients had partners who were also living with HIV and thus had disclosed to them. The high rate of HIV disclosure among clients is likely due to the clinic’s care protocol encouraging clients to inform someone in their life about their HIV status to get support from them.

Many clients who reported disclosing their status shared that they felt accepted or were not stigmatized/discriminated against once they disclosed. As one client (FGD 2, R3) who had disclosed to her family shared, “In fact, my family also didn’t stigmatize against me. I was first in Accra where I was diagnosed. My sister was the one who brought me here and everything is normal, there has not been any stigma.”

Most PLWH clients who reported disclosing their status felt supported by those they disclosed to. The form of support most discussed by PLWH was treatment support such as reminders to take their medication. One client shared that,

“In my house, my grandmother, my mother’s mother is aware. My uncle is aware. My father is aware. All my seven children are aware. So, we all live together. It was even my second child who brought me to the hospital because the eldest child is not living with me, and he called the fourth born who is my eldest daughter. She also came and they got together. So, all of them [ask], ‘Mama, have you taken your drug? Mama, have you done that?’ So, in my house, everybody knows.” (FGD 4, R2)

Some participants who disclosed their status were related to or in relationship with someone who were also living with HIV. One such client explained how her partner supports her and her HIV-positive child in taking their treatment medications and defying internalized HIV stigma.

“So, when we were [leaving clinic], he also said that there are some sicknesses that are more serious than this one…in my case he’s the only one I told…He has rather been encouraging me that I should be coming for the medication on time. The nurses have counseled him about the thing so once he understood it, he himself has been reminding us [wife and child] about our medication.” (FGD 4, R)

Additional forms of support clients discussed included emotional support, including encouragement they received from their spouses, children, and HCWs after disclosing their status. Some clients particularly wanted to isolate from their family, not be in a partnership after they learned of their status, and/or felt disheartened due to HIV stigma. With emotional support from their loved ones, they reintegrated with their family and accepted their situation. For example, one client (FGD 2, R2) who wanted to “run away” and leave her relationship after testing positive recalled how her husband, who was HIV negative, consoled her about her HIV status and reassured her that they could still be sexually intimate with the right precautions. Other people reported receiving instrumental support such as financial support and support with house chores when they were too sick to work or do chores. One client who had only disclosed to her son shared how he supported her with chores and job tasks when she did not have the strength to do either,

“When it started, I couldn’t do anything, so he was the one who did all the chores. But now me myself am strong so he doesn’t do anything. But when I go to the roadside, those selling stuff, he does transfers just close to me, so he sells some of the things for me…When I’m feeling sleepy then I sleep for a while then he’ll be selling. When I wake up then I continue.” (FGD 4, R5)

## Discussion

To date, only a few studies have examined the impact of stigma among PLWH in Ghana. Our study thus contributes quantitative data and qualitative descriptions of PLWH’s experiences with different forms of HIV-related stigma and their impact on HIV treatment and care in Ghana. Both the qualitative and the quantitative findings from the present study suggest that PLWH fear and experience stigma, which is impacting their HIV treatment and care-seeking behavior, as well as their psychosocial wellbeing.

From the quantitative findings, the association of stigma and social support are seemingly paradoxical. In the bivariate analysis, we found that internalized stigma is associated with higher support, thus, those who reported low internalized stigma had low social support. Similarly, among those with low internalized stigma, almost a third reported high social support while among those with high internalized stigma, two-thirds reported high social support. In multivariate analysis, we found that high social support was associated with 15 times greater odds of having high internalized stigma which was also confirmed by the qualitative findings. This finding is consistent with an earlier finding among women living with HIV in Uganda [31] which found that PLWH were more likely to report high internalized stigma. A possible explanation of this finding is that the PLWH who have high social support are likely to have more people in their social circle and may therefore encounter and internalize more experiences that are stigmatizing or discriminatory. The findings suggests that while social support is important in helping PLWH navigate life and adhere to treatment, care must be taken in their social networks to ensure that they are supportive, rather than contributing to experiences of stigma. Another possible explanation emerges from our qualitative findings, which indicate that family members and care providers provided social support, particularly emotional support in response to clients internalized HIV stigma. Thus, the positive association between high internalized stigma and high social support in quantitative findings may be because clients with internalized HIV stigma may be receiving more emotional and affirming support from family to console and encourage them about their status. Longitudinal studies on the relationship between social support and internalized HIV stigma can provide stronger data on the directionality of this relationship.

Consistent with prior studies [31–33], results in our study also indicate that none of those who had low internalized stigma reported depression symptoms, while those with high stigma reported more depressive symptoms, suggesting that stigma is associated positively with depression. Previous studies have reported associations between HIV-related stigma and negative health outcomes specifically [34–37]. Participants in our study also described how stigma impacted their mental health and engagement in care. These findings underscore the importance of consideration of HIV-related stigma in depression treatment for PLWH and contribute to the sparse studies on stigma and depression in Ghana.

Another unexpected finding from our study is that the majority of the participants who reported low internalized stigma reported non-adherence to the ART compared to those who reported high internalized stigma. This finding is consistent with prior studies which reported that HIV-related stigma undermined ART adherence by compromising general psychological processes, such as adaptive coping and social support [10]. While qualitative findings did not provide direct insights into why participants with low internalized stigma had high ART non-adherence rates, we did find that internalized and anticipated stigma influenced people to avoid HIV care, which seemingly contradicts this quantitative finding. One possible explanation based on the FGDs is that PLWH who experience internalized stigma are motivated to adhere to their ART in order to avoid the worsening of the illness, which may disclose them as ill. It would be helpful for future research to further explore this relationship between stigma and ART adherence among PLWH.

The qualitative findings provide more in-depth understandings of forms and effects of HIV stigma experienced by PLWH in addition to the quantitative findings on factors associated with internalized stigma and impact of internalized stigma on ART outcomes. Particularly, in qualitative interviews with clients and service providers, we observed that anticipated stigma was the most reported concern of PLWH clients, followed by internalized and enacted stigma. Clients experienced these forms of stigma on the individual, interpersonal, community, organizational, and societal levels.

Healthcare providers also described how to support and provide a safe space for their clients to adhere to ART and engage in care. Future research should focus on understanding how resilience can be used to respond to or counter the negative effects of HIV-related stigma in order to improve HIV treatment outcomes.

Sources of participants’ anticipated or encountered stigma included healthcare settings and health professional, community settings, at HIV programs/interventions, and from friends/acquaintances, family, PLHW peers, community members, and healthcare providers and staff. These findings are consistent with Rice and colleagues’ study [38], which found that enacted and anticipated HIV-related stigma in healthcare settings were associated with poor ART adherence. Consistent evidence corroborates that anticipation and enacted stigma in healthcare settings inhibits engagement along the continuum of HIV prevention and care [38–40]. The stigma anticipated and experienced in clinical sites and with other PLWH may be shaping the stigma anticipated in HIV interventions. As researchers, we assume clients will be helped by treatment and care support interventions, yet we found in our interviews that there were concerns and fears of participating in potential interventions due to experienced and internalized stigma. We thus need to take these concerns about stigma into consideration in how we design and implement interventions to mitigate clients’ fears and concerns about experiencing stigma in treatment and care support interventions and to maximize participation in these programs.

The high number of clients reporting HIV disclosure to family members in qualitative interviews may help explain why in quantitative findings, HIV disclosure was not associated with internalized stigma. It is possible that clients are disclosing their status regardless of internalized stigma due to HIV providers’ encouraging them to disclose to a loved one as recommended in treatment and care protocols.

While there was no statistical significance between HIV status disclosure and treatment outcome, the qualitative results highlighted the negative experiences associate with serostatus disclosure and treatment outcome. Importantly, fear of experiencing stigma is found to be a major factor preventing PLWH from disclosing their serostatus to partners and family members. This is consistent with other studies which found that PLWH who disclose their HIV status experience stigma from their family compared to those who do not disclose their status [37, 41–43]. This highlights the importance of designing multilevel stigma reduction interventions that not only focus on PLWH but also on their family, partners, and community.

The results of this study should be interpreted considering several study limitations. First, it was a cross-sectional study; therefore, we cannot assume causality between internalized stigma, depression, and adherence to ART. Furthermore, our sample size was relatively small, thus affecting our power to detect all associations of non-adherence. Moreover, because we recruited participants from a single clinic in the Volta region of Ghana, we are unable to generalize our findings to those outside this region, nonetheless, the findings are compelling enough to inform similar studies on a national scale. We recommend that future studies use larger randomly selected patient samples to improve internal and external validity. It is also important to note that we only measured internalized stigma in the survey, therefore, we do not have quantitative data on the other forms of stigma. However, the focus group discussions among the participants have provided rich information on their experiences with the other forms of stigma. Another limitation was the challenge with the large amount of missing data for some variables, which has a potential to bias our results if missing not at random (MNAR). Finally, our study used self-reported measures, which may be prone to social desirability bias given the sensitive nature of the topics of ART, stigma, and depression. This bias may also lead to an underestimation of the true effect of these factors.

## Conclusion

This study contributes to the literature on HIV stigma, psychosocial, and HIV treatment outcomes among PLWH. Findings suggest an urgent need for HIV stigma reduction interventions for PLWH, their family and friends, healthcare providers, and community members. Based on four findings, PLWH might benefit from stigma intervention with a peer support component. Future studies can explore the national, regional, and sub-regional variations of the relationship between HIV-related stigmas, psychosocial factors, and ART adherence among PLWH in Ghana.

## Supporting information

S1 Text PLoS inclusivity in global research checklist. (DOCX)
